# Click Chemistry for the Synthesis of RGD-Containing Integrin Ligands

**DOI:** 10.3390/molecules15010178

**Published:** 2010-01-06

**Authors:** Matteo Colombo, Aldo Bianchi

**Affiliations:** 1 NiKem Research, via Zambeletti 25, 20021 Baranzate, Milan, Italy; 2 Consorzio Interdisciplinare di Studi Biomolecolari ed Applicazioni Industriali (CISI), Via Fantoli 16/15, 20138 Milan, Italy; E-Mail: aldo.bianchi@cisiscrl.com (A.B.)

**Keywords:** click chemistry, RGD, pseudopeptide, biomaterials, integrins

## Abstract

In the last few years click chemistry reactions, and in particular copper-catalyzed cycloadditions, have been used intensively for the preparation of new bioconjugate molecules and materials applicable to biomedical and pharmaceutical areas. This review will be focused on conjugates of the tripeptide Arg-Gly-Asp formed by means of click chemistry reactions. This sequence is a well known binding motif for specific transmembrane proteins and is involved in cellular adhesion to the extracellular matrix, allowing the selective recognition of the biomolecule or polymer in which it is incorporated.

## 1. Introduction

“*The reactions must be modular, wide in scope, give very high yields, generate only inoffensive byproducts that can be removed by nonchromatographic methods, and be stereospecific. The required process characteristics include simple reaction conditions (ideally, the process should be insensitive to oxygen and water), readily available starting materials and reagents, the use of no solvent or a solvent that is benign (such as water) or easily removed, and simple product isolation.*” These are the set of general criteria indicated by Kolb, Finn and Sharpless in 2001 to define a “click chemistry” reaction [1].

Carbon-heteroatom bond forming reactions and in particular cycloaddition reactions, nucleophilic ring-opening reactions, carbonyl chemistry of the “non-aldol” type, and additions to carbon-carbon multiple bonds are the typical examples of these kind of near-perfect chemical transformations.

**Scheme 1:**
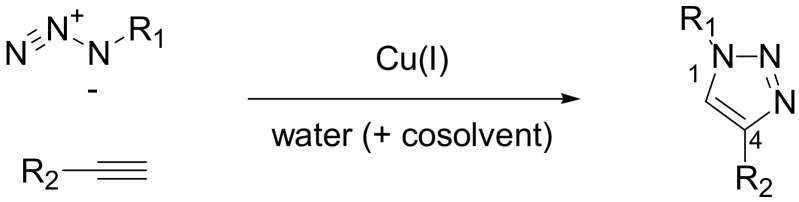
Copper-catalyzed alkyne-azide cycloaddition (CuAAC).

Among the aforementioned type of click transformations, undoubtedly, the premier click chemistry reaction in terms of applications and spread is the Cu(I)-catalyzed alkyne-azide cycloaddition (CuAAC). This copper-catalyzed version of the Huisgen 1,3-dipolar cycloaddition demonstrates all the necessary characteristics of a click reaction: prompt accessibility to starting materials, running and purification simplicity, very high yields, complete regioselectivity and widespread applicability (Scheme 1). For these reasons it is often defined erroneously as the “click chemistry” reaction. Moreover, 1,4-substituted 1,2,3-triazoles, the products of CuAAC, are able to function as rigid linking units that mimic the atom placement and electronic properties of an amide bond without the same susceptibility to enzymatic hydrolytic cleavage [2].

Since 2001 a plethora of papers involving click reactions has been published in various areas of synthetic chemistry, including drug discovery, biomolecules, macromolecules and materials. In this review we would like to focus our attention on recent advances of the applications of click chemistry reactions for the synthesis of molecules and materials bearing the tripeptide arginine-glycine-aspartic acid (Arg-Gly-Asp or RGD).

**Figure 1:**
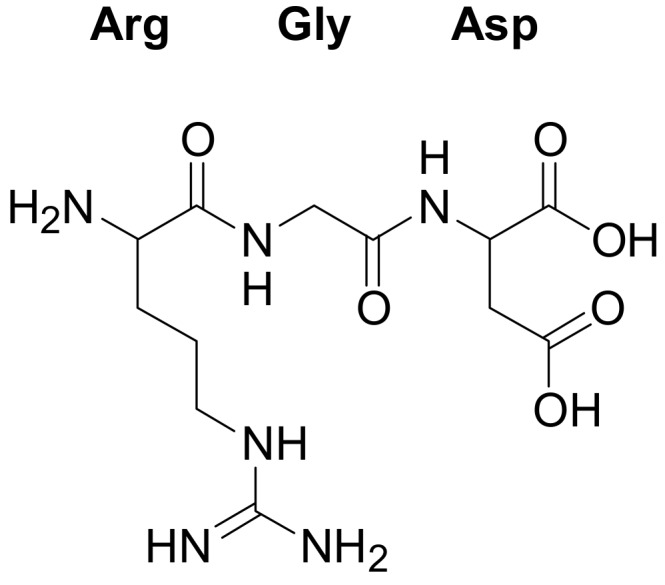
Tripeptide Arg-Gly-Asp.

The RGD sequence (Figure 1) has been identified as an essential binding motif for at least seven integrin receptors [3,4]. Integrins are heterodimeric (α−β) transmembrane proteins expressed at the cell surface and involved in cellular adhesion to the extracellular matrix [5,6]. They stimulate vascular endothelial cell migration and invasion, regulating their growth, survival and differentiation and they serve as receptors for a variety of extracellular matrix proteins including vitronectin, fibronectin, fibrinogen and osteopontin. They are involved in many biological processes such as angiogenesis, thrombosis, inflammation, osteoporosis and cancer playing a key role in many severe human diseases [7,8,9]. Up until now, 18 α and 8 β subunits have been identified: they form 24 heterodimers, each with distinct ligand binding properties.

Among the integrin superfamily, α_v_β_3_ and α_5_β_1_ integrins, targeted by the RGD sequence, play a pivotal role in the formation of new blood vessels in tissues (angiogenesis). They are overexpressed on activated endothelial cells during physiological and pathological angiogenesis [10]. Since α_v_β_3_-integrin is expressed on tumor cells of various types (melanoma, glioblastoma, ovarian and breast cancer) where it is involved in the processes that govern metastasis, it represents an attractive target for cancer therapy and has stimulated ongoing research to define high affinity ligands [11,12]. RGD-containing integrin ligands have a large number of medical applications ranging from noninvasive visualization of integrin expression *in vivo*, to the synthesis of functionalized biomaterials.

This review will be divided into two different sections and cover the literature up until November 2009. The first section will discuss the synthesis of pseudopeptides containing the aforementioned tripeptide, whereas the second part will be focused on the introduction of the RGD sequence into polymers and biomaterials. The greater number of reported examples will be based on CuAAC chemistry, though some interesting applications of alternative click transformations will be illustrated.

## 2. Functionalized Pseudopeptides

The introduction of the RGD sequence in appropriately functionalized pseudopeptide structures allows the targeting of specific overexpressing-integrin cells, transferring to the biological system the peculiar characteristics of the synthesized peptides.

An important application which has recently received increasing interest is the preparation of probes for noninvasive visualization of the α_v_β_3_-integrin expression status. Various imaging modalities are available, ranging from nuclear imaging (SPECT: single electron emission computed tomography, PET: positron emission tomography), over magnetic resonance (MRI: magnetic resonance imaging), to optical methods dyes (FMT: fluorescent mediated tomography). All these methods require an integrin targeting probe conjugated to a suitable signal unit, that is, a radionuclide, a contrast agent or a fluorescent dye [13].

As the following examples will show, the click chemistry approach for the preparation of pseudopeptide compounds, both used as biological probes and substitutes of natural peptides, allows the synthesis of large biological molecules using mild reaction conditions. Often the protection of delicate functional groups is not required and a simple purification step affords the desired biomolecules, in a suitable state for biological assays.

### 2.1. Radiohalogenated compounds (^18^F-labelled compounds)

**Scheme 2:**
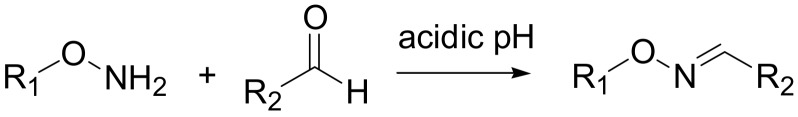
Chemoselective oxime ligation.

Chemoselective oxime ligation, a selective reaction of an aldehyde with a hydroxyl amino group (see Scheme 2), has been proposed by Kessler and co-workers as a versatile tool for the introduction of a radio marker in peptides. The major advances of this methodology are the high chemoselectivity, the use of unprotected aminooxy precursors and the fact that the coupling with the carbonyl component can be performed in aqueous media.

**Figure 2:**
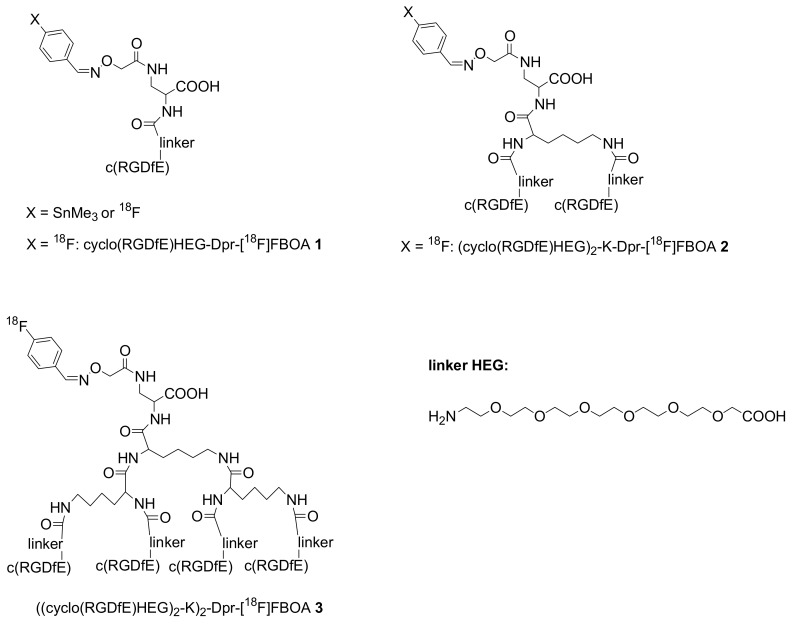
Structures of [^18^F]FBOA labelled cyclo-RGD peptides.

**Scheme 3:**
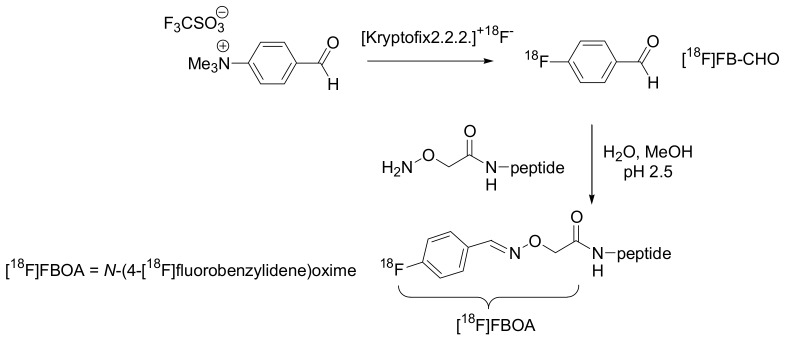
Radiosynthesis of [^18^F]FB-CHO followed by coupling to aminooxy-functionalized peptides *via* oxime formation.

Kessler described in detail the solid phase synthesis of peptides containing the cyclo-RGDfE sequence, appropriately linked to the aminooxy functionality required for the chemoselective oxime ligation. The subsequent coupling of these peptides with *p*-trimethylstannylbenzaldehyde afforded useful precursors for radioiodostannylation (Figure 2) [14]. The advantages of this methodology were then used by the same authors for the efficient synthesis of ^18^F-labelled peptides [15]. ^18^F is an important source of positrons and it represents the ideal radionuclide for PET. ^18^F-labelled target specific peptides are widely used as *in vivo* imaging agents but, because of the physical half-life of ^18^F (109.7 min), it is very important to have in hand a synthetic method which allows the clean and rapid introduction of the radio label. 4-[^18^F]Fluorobenzaldehyde ([^18^F]FB-CHO) was prepared from the 4-formyl-*N*,*N*,*N*-trimethylanilium precursor *via* direct no-carrier-added ^18^F-fluorination and, after purification, it was coupled to aminooxy-functionalized peptides with an overall radiochemical yield (decay corrected) of up to 40% (Scheme 3).

The chemoselective oxime ligation has been applied for the preparation of ^18^F-labelled multimeric cyclo-RGDfE peptides, as depicted in Figure 2. There is a clear positive effect for multivalency: the affinity of **1** *vs.* **2** *vs.* **3** for the α_v_β_3_-integrin increased by around a factor of 10 with each multimerization step leading to improved tumor accumulation.

Peptide **2**, as a representative RGD analogue, was used for extensive *in vivo* biodistribution studies showing favorable pharmacokinetics and high tumor-to-non-tumor ratios, thus demonstrating the suitability of these labelled peptides for PET applications.

**Scheme 4:**
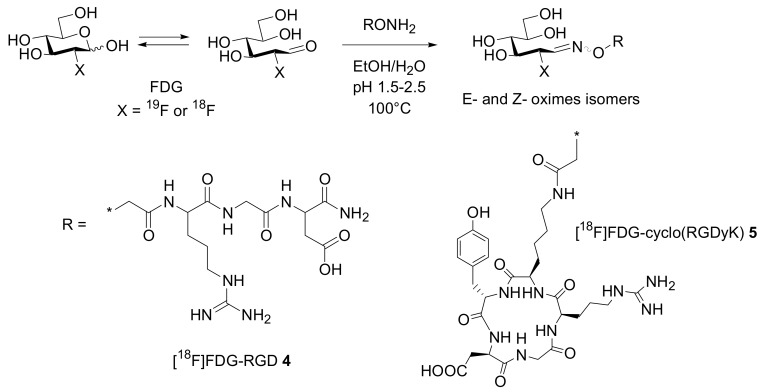
Synthesis of **4** and **5** by chemoselective oxime ligation.

However, using 4-[^18^F]-fluorobenzaldehyde as the prosthetic group invariably increases ligand lipophilicity, which often leads to a deterioration in tracer pharmacokinetics. To prevent this effect in two recent publications cited the use of the well accepted and easily available 2-[^18^F]-fluoro-2-deoxyglucose ([^18^F]FDG) PET tracer as a prosthetic group for the synthesis of ^18^F-labelled peptides. Gambhir and co-workers synthesized two labelled RGD-containing peptides **4** and **5** *via* chemo-selective oxime ligation [16]. At 100 °C, FDG exists in a dynamic equilibrium between its cyclic and linear form. This second form contains the aldehyde functionality that can react with the aminooxy functionality of a suitably modified peptide. Linear- and cyclo-aminooxy-RGD peptides were reacted at 100 °C with FDG in the presence of 0.4% TFA to give the corresponding conjugates in a 27.5 and 41% radiochemical yield (decay corrected) respectively (Scheme 4). High-contrast PET images with relatively moderate tumor uptake were observed for the two compounds as PET probes in xenograft models expressing α_v_β_3_ integrin.

**Scheme 5:**
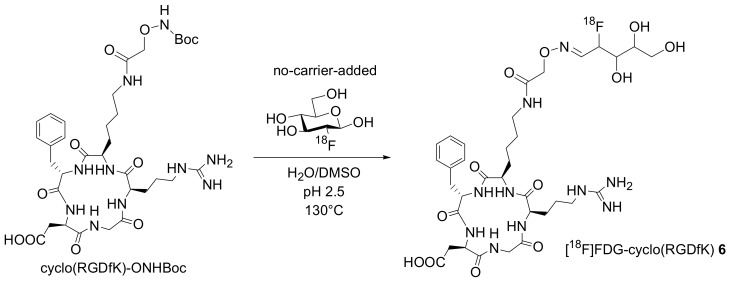
Synthesis of [^18^F]FDG-RGD **6** by chemoselective oxime ligation.

Analogously, Wester and co-workers described the direct conjugation of an aminooxy-functionalized RGD peptide cyclo(RGDfK)-ONH_2_ with no-carrier-added [^18^F]FDG [17]. No-carrier-added [^18^F]FDG was obtained by HPLC separation from excess glucose of the [^18^F]FDGTUM prepared from routine in-house synthesis at the Technische Universität München (TUM). The corresponding conjugate was obtained in yields of up to 93% (decay corrected) and activities up to 37MBq (Scheme 5).

In a preliminary *in vivo* biodistribution study in M21 melanoma-bearing nude mice, [^18^F]FDG-RGD showed increased tumor accumulation compared to [^18^F]-galacto-RGD, the only integrin-ligand presently in clinical studies [13].

**Scheme 6:**
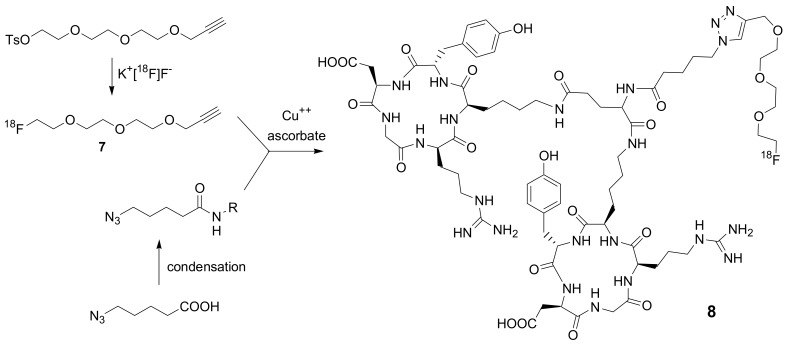
Radiosynthesis of ^18^F-fluoro-(triethylene glycol)-triazole-E[c(RGDyK)]_2_ **8**.

A different synthetic approach for the introduction of the prosthetic group was employed by the group of Chen. In this case, the labelling reaction is based on the Cu(I)-catalyzed Huisgen 1,3-dipolar cycloaddition. The authors prepared a dimeric-cyclo-(RGDyK) peptide labelled with their newly developed ^18^F synthon as represented in Scheme 6 [18]. Nucleophilic fluorination of the *p*-toluenesulfonic alkyne provided ^18^F-alkyne **7** in high yield (65% nondecay-corrected yield) and under mild conditions. The presence of the triethylene glycol linker is necessary to reduce volatility and to obtain water solubility. The azide moiety was introduced on the biomolecule by condensing the glutamate amino group of the cyclo-(RGDyK) dimer with an opportune azide-activated ester. The 1,3-dipolar cycloaddition is an extremely useful method for the introduction of the ^18^F synthon, considering both the short reaction time (45 minutes including HPLC purification) and the high labelling yield (52 ± 8% nondecay-corrected yield). The ^18^F-labelled peptide **8** was subjected to microPET studies in murine xenograft models showing good tumor uptake and relatively good metabolic stability as well as favourable *in vivo* pharmacokinetic.

### 2.2. Radiometalated compounds

**Figure 3:**
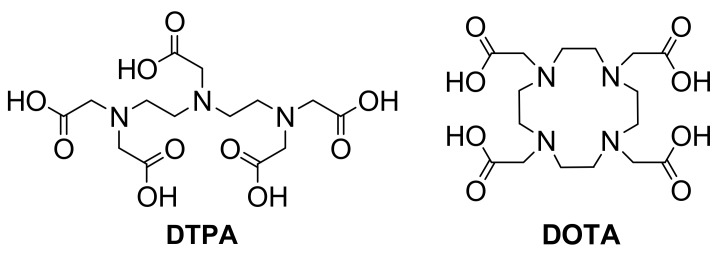
Bifunctional radiometal chelating systems.

A different approach for potential tumor targeting and imaging involves the use of bifunctional radiometal chelating systems such DOTA (1,4,7,10-tetraazacyclododecane-1,4,7,10-tetraacetic acid) or DTPA (diethylenetriaminepentaacetic acid) (Figure 3).

**Figure 4:**
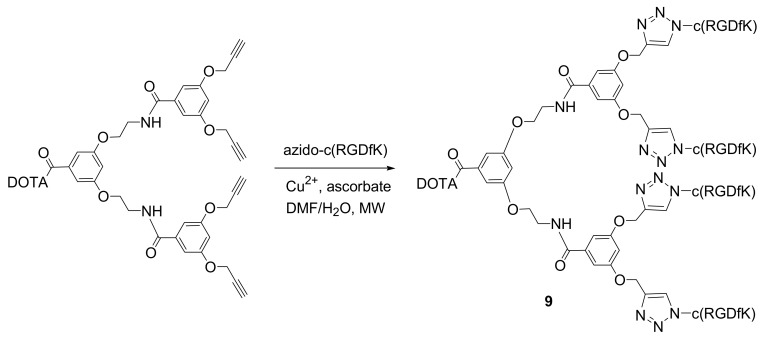
DOTA conjugated tetravalent cyclo(RGDfK) peptide dendrimer **9**.

Some α_v_β_3_-integrin ligands have been radiometalated using chelators of this type. One example comes from the group of Liskamp where a series of multivalent cyclo-RGDfK peptides were conjugated to DOTA *via* 1,3-dipolar cycloaddition [19]. A convergent synthesis of aminoacid-based dendrimers was employed for the preparation of DOTA conjugated dendrimeric alkynes. They were subsequently coupled *via* a microwave assisted 1,3-dipolar cycloaddition to azido cyclo-RGDfK peptides. The representative structure of the tetravalent derivative **9** is shown in Figure 4. It should be emphasized that the carboxylic functionality of the DOTA-moiety needed to be protected by *t*-butyl groups during the cycloaddition step to avoid premature and irreversible sequestering of Cu^2+^ ions. Chelated copper (II) results in a lower efficiency of the redox couple and it hampers the radiolabeling of DOTA-moiety with trivalent radiometals such as ^111^In, ^90^Yt or ^177^Lu. Thus, after the click reaction an additional step of deprotection is required.

All the synthesized analogs were radiolabelled with ^111^In to evaluate the *in vitro* receptor binding characteristics and *in vivo* tumor targeting properties. IC_50_ measurements showed the enhanced affinity effect of multivalency, while biodistribution studies showed that the tetrameric RGD-dendrimer possessed better tumor targeting properties than its dimeric and monomeric congeners.

**Scheme 7:**
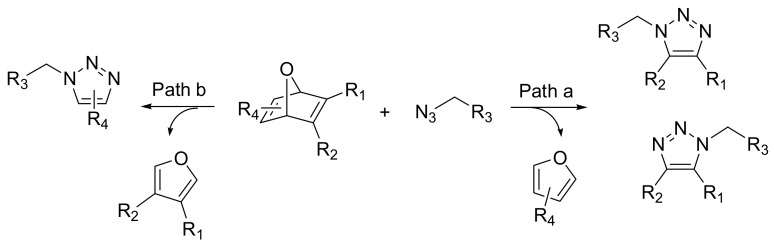
Reaction pathways for the metal-free triazole formation.

Copper-independent strategies have been developed for *in vivo* use. Rutjes and co-workers recently described how easily accessible trifluoromethyl-substituted oxanorbornadiene derivatives react with azides in a tandem [3+2] cycloaddition-retro-Diels-Alder reaction (crDA) to form stable triazole-linked compounds. As this reaction can be performed in aqueous media, at ambient temperature, and in absence of copper, it represents a valid alternative to the classical Huisgen azide-alkyne cycloaddition. Moreover, oxanorbornadiene derivatives possess an increased reactivity towards azides compared to the corresponding electron-deficient alkynes. Although both double bonds can react, the 1,4,5-substituted triazoles were predominantly formed (Path a, Scheme 7). As a first example of the application as a new bioconjugation method for the functionalization of peptides and proteins, an azide-functionalized hexapeptide (GGRGDG) was conjugated to a PEG functionalized oxanorbornadiene [20].

**Scheme 8:**
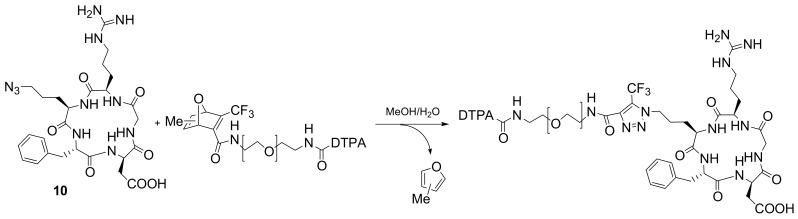
Tandem crDA reaction of c(RGDfX) and methyloxanorbornadiene-DTPA.

The absence of copper during the conjugation step is particularly useful for the introduction of ligands for radiolabelling such as DTPA. A DTPA radiolabel was conjugated to a *N*-δ-azido-cyclo(RGDfX) **10** *via* oxanorbornadiene derivatives (Scheme 8) [21]. Preliminary biological evaluation showed high affinity for integrin α_v_β_3_ (IC_50_ = 192 nM) and favourable pharmacokinetics.

The effects of the concentration of the reactants, temperature, pH and reaction environment on the kinetics of the reaction were then evaluated in order to optimize the synthesis. The biodistribution of the labelled compound in mice (^111^In was selected because of its long half-life: 67.2 h and its stability in the DTPA chelate) was determined, confirming specific accumulation of the conjugate in α_v_β_3_-integrin-expressing tissues [22].

### 2.3. Fluorescent compounds

**Scheme 9:**
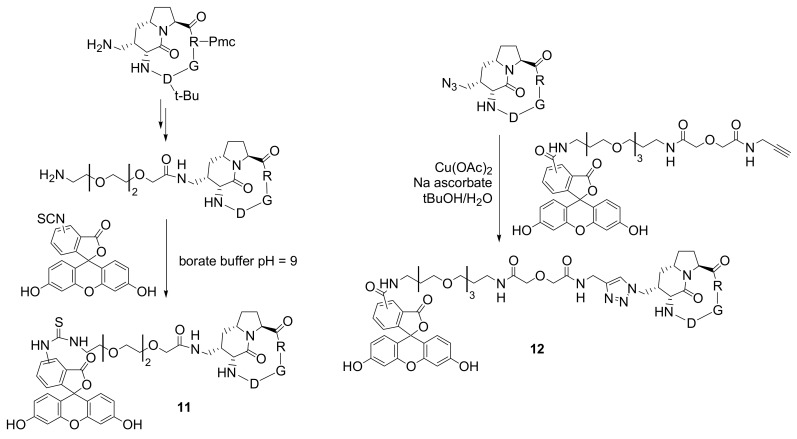
Synthesis of cyclo-RGD peptide-fluorescein conjugates **11** and **12**.

Another important method used for *in vivo* imaging is the introduction of fluorescent dyes. Manzoni and co-workers have recently reported the synthesis of two cycloRGD peptide-fluorescein conjugates **11** and **12** that could be used as *in vivo* markers for α_v_β_3_-integrin expression in human cells [23]. The green fluorescent dye fluorescein is widely used as a probe due to its known safety profile. Two different synthetic strategies were used by the authors for the conjugation with fluorescent probes of cyclic RGD compounds containing the conformationally constrained homoSer-Pro dipeptide unit. The first strategy involved the functionalization of the RGD cyclopeptide with a short poly(ethylene glycol) spacer armed with a terminal amine group and able to form a stable thiourea bridge with commercially available fluoresceine isothiocyanate, through an efficient click carbonyl chemistry reaction. The second approach required the preparation of a fluorescent derivative with a terminal triple bond that can be directly reacted with the azide group of the RGD cyclopeptide through the copper-catalyzed 1,3-dipolar cycloaddition (Scheme 9).

After *in vitro* treatment, the fluorescent probe was detectable in a panel of cell lines highly expressing α_v_β_3_ integrin as endothelial cells (HUVEC) and several human cell lines derived from solid tumors. Moreover, the pattern of detected fluorescence also suggested that both compounds were dynamically internalized by cells.

**Figure 5:**
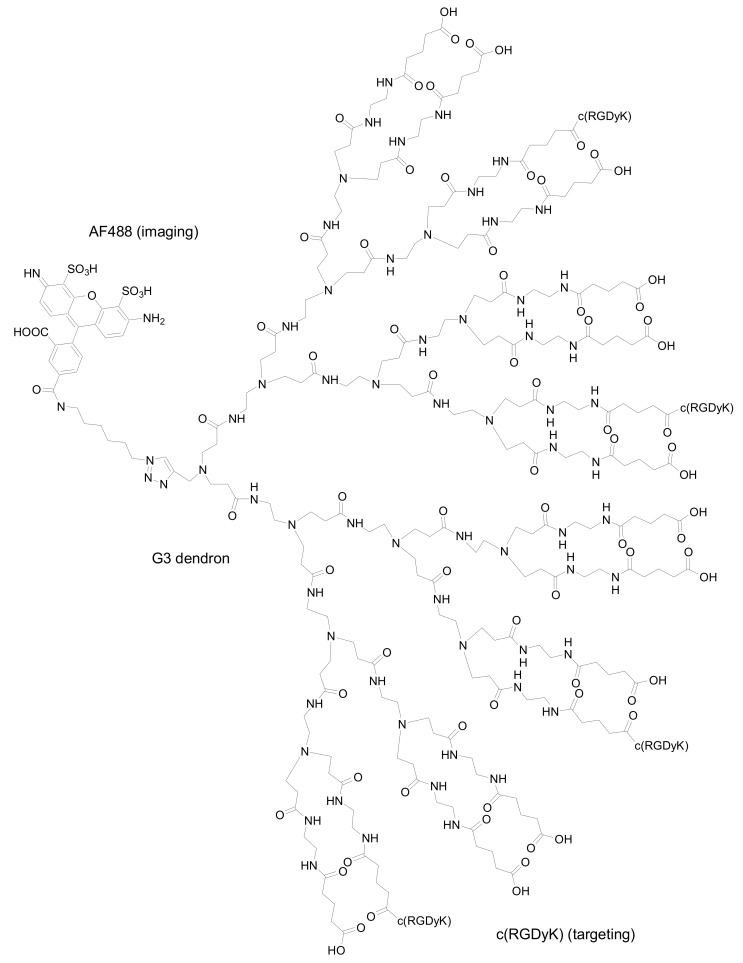
Structure of the fluorescent dendron conjugate AF-G3(COOH)_12_(RGD)_4_.

In another recent example, Baker and co-workers described the synthesis and evaluation of *in vitro* biological activity of poly(amidoamine) (PAMAM) dendrons with c(RGDyK) peptides on their surfaces. A unique alkyne group present at the dendron focal point was reacted with an azide-functionalized dye [derivatized Alexa Fluor (AF) 488] *via* 1,3-dipolar cycloaddition thus generating a fluorescent high avidity binding agent (Figure 5) [24]. AF488 was chosen as the fluorescent label for the detection of conjugates as it is brighter than fluorescein and more resistant to photobleaching. The specific binding of the conjugate to α_v_β_3_ integrin expressing HUVEC and U87MG cells was examined by flow cytometry. The alkyne functionalized dendron was also conjugated to biotin, to a second dye-conjugated dendron and to methotrexate, a therapeutic drug, using the appropriate azide-derivatized molecule.

### 2.4. Peptide analogues

**Scheme 10:**
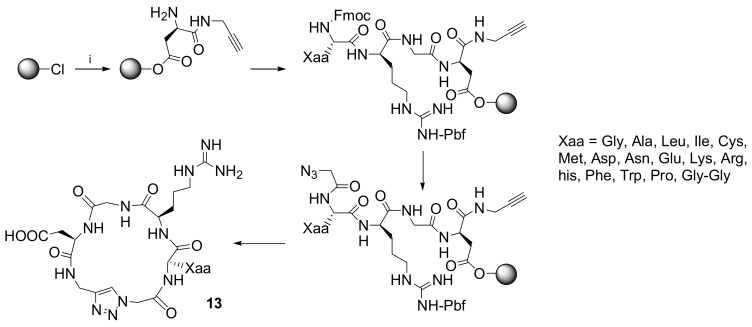
Synthesis of cyclopeptide analogues cyclo[-Arg-Gly-Asp-ψ(triazole)-Gly-Xaa-] **13**.

Click chemistry has been recently employed for the preparation of peptide analogues after the application of the 1,2,3-triazole moiety was reported as peptide bond isosteres. A new family of cyclopeptide analogues cyclo[-Arg-Gly-Asp-ψ(triazole)-Gly-Xaa-] **13** in which an amide bond was replaced by the 1,2,3-triazole moiety was synthesized by Pan and co-workers [25]. The synthetic strategy described in this paper represents the first example of the application of the [3+2] Huisgen cycloaddition in solution cyclization and synthesis of integrin binding peptides as outlined in Scheme 10.

The linear peptide analogue (N_3_-Gly-Gly-Arg(Pbf)-Gly-Asp-propargyl) was synthesized by standard solid-phase peptide synthesis strategy on resin. The cyclization was performed in solution and a variety of reaction conditions were evaluated to optimize the process. The best results were obtained employing 1/3 mol per cent of CuBr/DBU as the catalyst and dichloromethane as the solvent. The cytotoxic activity of all the cyclopetide analogues was evaluated using MTT method, showing, in some cases, activities comparable to cRGDfK that was employed as the positive control.

**Scheme 11:**
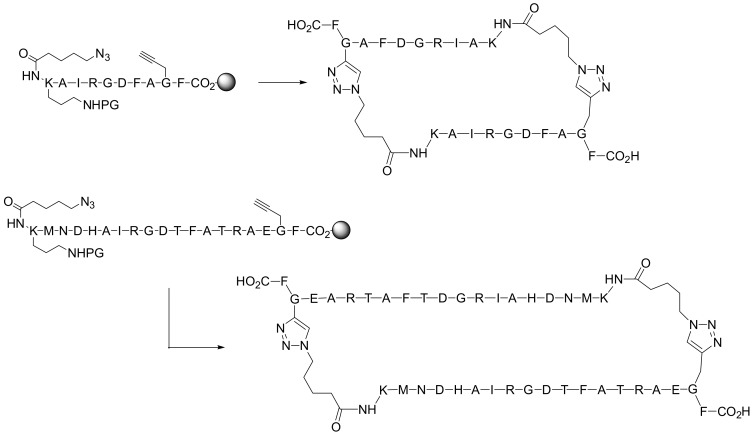
On resin cyclodimerization.

In a second example, Finn and co-workers described the selective production of large cyclic dimers from resin-tethered peptides containing azide and alkyne residues. The 11-mer and 19-mer RGD-containing peptides shown in Scheme 11 contain a sequence taken from adenovirus serotype that binds several α_v_ integrins. Head to tail macrocyclization was performed *via* 1,3-dipolar cycloaddition using 0.5 equivalent of the Cu(I) catalyst (Scheme 11) [26]. The properties of the resin and the solvent were found to be crucial for the selectivity of the process (cyclic dimers *vs.* cyclic monomers). The process resulted independent of the peptide sequence but the need for a long and flexible chain was confirmed by the fact that peptides shorter than hexamers were predominantly converted to cyclic monomers [27].

### 2.5. Glycosylated peptides

**Scheme 12:**
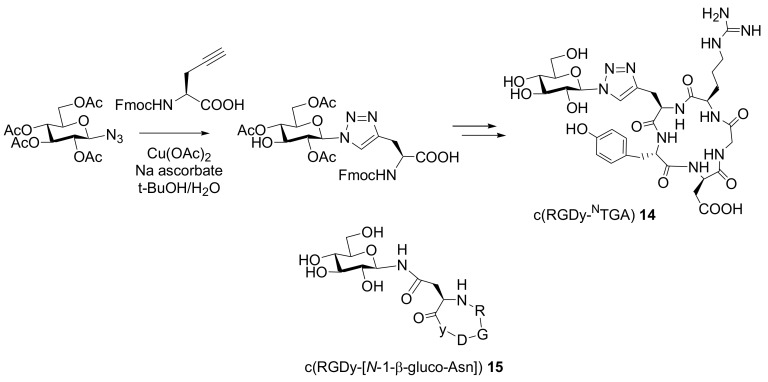
Synthesis of glycosylated amino acid and c(RGDy-*^N^*TGA) **14**.

The preparation of a glycosylated RGD derivative in order to evaluate the value of a triazole moiety as an amide isostere in terms of electronic properties and atom placement has been recently described by Rutjes and co-workers [28]. The authors showed that 1-azido sugars and acetylene-modified amino acids readily undergo [3+2] cycloaddition to form triazole isosters of glycoamino acids (Scheme 12). The obtained glycosylated amino acid was used as a building block in the following solid phase synthesis of the cyclic peptide c(RGDy-*^N^*TGA) **14**.

The presence of the triazole analogue increased the chemical stability of the compound with respect to the amide linked c(RGDy-[*N*-1-β-gluco-Asn]) **15**. Moreover, biodistribution studies indicated that the glycosylated peptide showed improved pharmacological properties compared to more hydrophobic/lipophilic peptides such as c(RGDyV).

## 3. Biomaterials

During the last few years the use of materials such as polymers in conjugation with active biological molecules has increasing success, with consequent demand of more and more defined structures. The growth of click chemistry has enriched and simplified the strategies of conjugation between materials and organic molecules, due to the ready availability of active building blocks, the possibility of performing the reactions at room temperature in aqueous media and almost complete conversions [29]. This part of the review will be focused on the linkage between materials and systems containing the tripeptide RGD, highlighting how the polymers may gain useful applications in biomedical areas. 

**Scheme 13:**
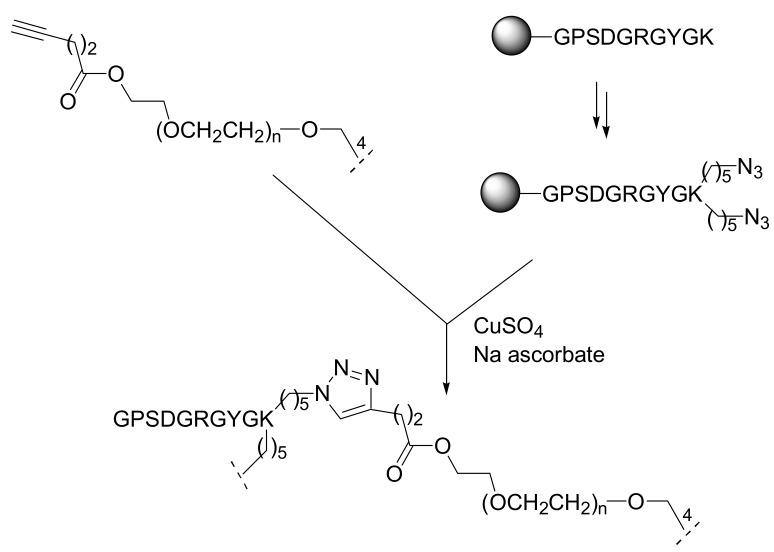
Synthesis of PEG-peptide hydrogel.

As first examples, hydrogels have been widely studied and utilized as biomaterials due to the high hydration level and the three dimensional scaffold structure, which give the similarities to the native extracellular matrix. Natural gelatin and collagen belong to this class of materials, but their use has been decreased because of the potential risk of infective diseases. Poly(ethylene glycol) (PEG), a hydrophilic polymer able to absorb large amounts of water and showing low immunogenicity represents a good substitute. Yang and co-workers prepared biodegradable PEG-peptide hydrogels containing the RGD sequence as potential cell delivery vehicles [30]. A stable triazole-based linkage between the polymer scaffold and RGD peptide was created by copper-mediated cycloaddition. Tetrahydroxy terminated 4-arm PEG was functionalized with 4-pentynoic acid, whereas a series of decapeptides were prepared by classical Fmoc-based solid phase peptide synthesis and then functionalized with 6-azidohexanoic acid. The subsequent CuAAC and gelation yielded the expected PEG-peptide hydrogels (Scheme 13). Cell attachment and proliferation experiments were performed to demonstrate the potential of PEG-RGD peptide hydrogels as useful biodegradable supports. Thanks to the integrin receptor-mediated extracellular signal, the cells on the hydrogels formed using a large amount of RGD (2.7 or 5.4 mM in the precursor solution) were well attached and spread well with healthy morphology thus making these materials promising carriers for cell delivery.

**Figure 6:**
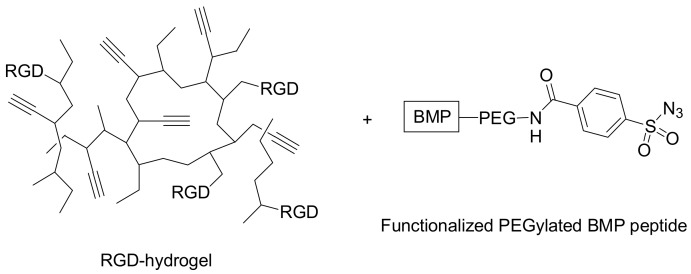
Schematic structures of RGD hydrogel and functionalized BMO peptide.

Analogously, Jabbari and co-workers took advantage of the combination of RGD peptides and hydrogel substrates to develop engineered scaffolds. The aim of the work was the grafting of the RGD peptide and BMP-2 (bone morphogenetic protein-2) peptide onto biodegradable inert hydrogels, and their use as scaffolds for the regeneration of skeletal tissues through osteogenic differentiation and mineralization of bone marrow stromal (BMS) cells [31]. The hydrogel scaffold, shown in Figure 6, was prepared by the cross-linking of macromer poly(lactide-ethylene-oxide-fumarate) (PLEOF) with acrylamide-termined GRGD and propargyl acrylate. Thereafter the PEGylated BMP peptide functionalized with 4-carboxybenzenesulfonazide was grafted onto the RGD hydrogel *via* copper-mediated cycloaddition in ambient conditions. Due to the fact that all the reaction by-products had a high solubility in aqueous solution, the purification of the gel was performed successfully by simple washing. The density on the hydrogel surface of both peptides was good, as well as the increased extent of mineralization of the BMS cell, obtained by the synergistic action of RGD and BMP peptides.

Click chemistry reactions have been largely used also for the functionalization of self-assembled monolayers (SAMs). A SAM is an organized layer of amphiphilic molecules formed on a variety of inert surfaces, including glass, silicon, gold diamond and amorphous carbon. The amphiphilic layer terminates with a head group showing special affinity for a substrate. Due to the capacity of controlling surface properties, such as bioinertness or biomolecule presentation, SAMs have emerged as helpful tools in the study of biochemical interactions.

**Scheme 14:**
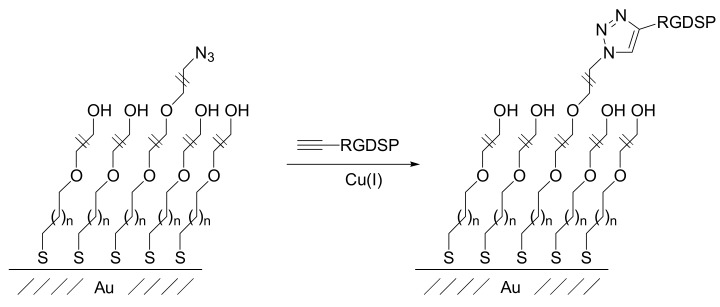
Conjugation of the RGD system on SAM *via* CuAAC.

Following this concept, Hudalla and Murphy prepared SAMs formed from the mixed coordination of different alkanethiolates onto a gold-coated substrate. In particular the authors used azide-terminated hexa(ethylene glycol) alkanethiolate and tri(ethylene glycol) alkanethiolate to prepare a SAM presenting groups to allow for a click chemistry conjugation. Immersion of the functionalized gold substrate into a solution containing the peptide Arg-Gly-Asp-Ser-Pro bearing a terminal alkyne moiety and the catalytic mixture CuBr and sodium ascorbate led rapidly and quantitatively to the chemoselective linkage of the peptide to SAM through the CuAAC reaction (Scheme 14). Terminal oligo(ethylene glycol) prevented nonspecific protein adsorption rendering bioinert the monolayer, however the presence of the RGD system allowed a selective adhesion and spreading of human mesenchymal stem cells, demonstrating how SAMs may be useful as active substrates for stem cell studies [32].

**Figure 7:**
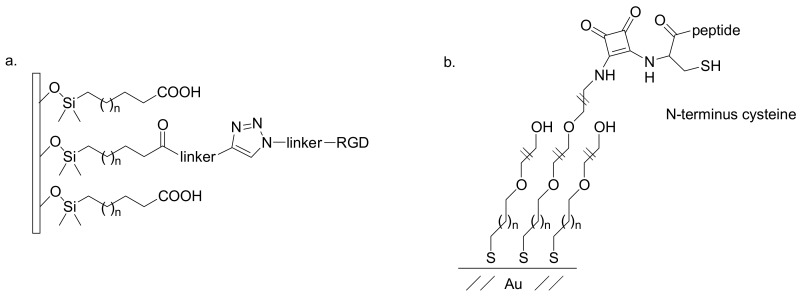
RGD-containing peptide immobilized onto silicon surface (a) and *N*-terminus cysteine immobilization on phenoxy-squarate SAM (b).

The RGD peptide was also introduced by Becker and co-workers onto SAM substrates by classical click alkyne-azide cycloaddition. The authors deposited n-octyldimethylchlorosilane onto silicon wafer and glass slide surfaces. A subsequent gradient ozone treatment produced selective terminal oxidation of the SAM which was then functionalized with a linker bearing a propargyl moiety. The RGD-containing peptide was covalently immobilized into the gradient substrate *via* copper-catalyzed triazole cycloaddition (Figure 7a). Smooth muscle cells were cultured on the layers and the number of adherent cells increased as a function of the position of gradient and hence of the increasing RGD density along the substrate, thus highlighting the ability of the SAM in modulating biofunctionalizations [33].

Luk and co-workers immobilized peptides onto alkanethiolate-based SAMs through an alternative substitution reaction, which can be considered click-like, due to the high chemoselectivity and irreversibility, easy purification and the use of water as the solvent. Terminal tri(ethylene glycol) groups of alkanethiol chains were functionalized with phenoxy squarate, which is able to couple covalently at pH 5.5 with *N*-terminus cysteine peptides selectively (Figure 7b). Peptides containing *C*-terminus cysteine and internal cycteines did not react under the reported conditions. To evaluate successful immobilization of the peptides on the surface of the SAM, the RGD sequence was introduced as a specific cell-adhesion assay [34].

The previuos examples have demonstrated that the incorporation of the RGD sequence onto a polymer or a substrate is a quick method to produce innovative materials able to promote cell adhesion and growth. The following examples will extend to the usefulness of click chemistry for other types of functionalized materials and in particular the use of CuAAC as a successful way to introduce the well-recognized adhesive RGD motif.

**Figure 8:**
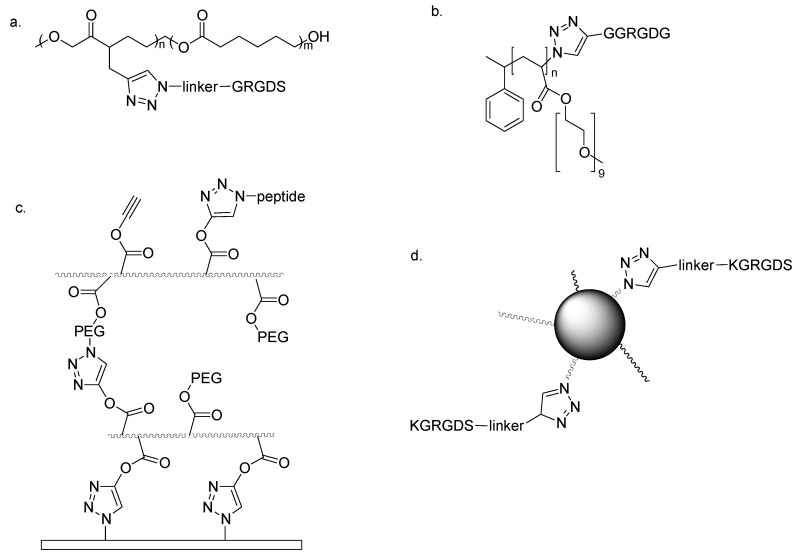
RGD systems on functionalized polymers: aliphatic polyesters (a), poly(oligo(ethylene glycol) acrylate) (b), PEG multilayer (c) and polymeric nanoparticles (d).

Emrick and co-workers prepared aliphatic polyesters with pendant acetylene moieties by tin-mediated ring-opening polymerization. Subsequently, PEGs or oligopepdides containing the RGD motif were grafted through click CuAAC (Figure 8a) [35]. An alternative polymer based on well-defined poly(oligo(ethylene glycol) acrylate) (POEGA) was prepared by Lutz’s group through atom transfer radical polymerization (ATRP). A bromine atom was present at the chain-ends, allowing a simple introduction of terminal azide moieties. Functionalized acetylenes, including the oligopeptide GGRGDG, were then anchored by efficient copper-catalyzed cycloaddition (Figure 8b) [36]. A further example is the peptide-functionalized multilayer prepared by Caruso and co-workers by the simple assembly technique known as layer-by-layer assembly. Each single PEG layer was covalently linked to the following through triazole moieties obtained by CuAAC. On the external surface of the modular PEG film free click groups were available for further cycloaddition reactions with appropriately decorated peptides, containing the RGD motif (Figure 8c) [37]. Niu, Wang and co-workers used M13 bacteriophage as a powerful nanoscale buiding block, thanks to the innate ability of this virus to organize into ordered films. The functionalization with spaced terminal alkynes of some amino groups on viral surface, known to be viable sites for chemical modifications, allowed the subsequent conjugation *via* click triazole synthesis of the RGD motif [38]. The last example reported here is the preparation of self-assembled polymeric nanoparticles, formed by the coupling of the hydrophobic poly(2-methyl-2-carboxytrimethylene carbonate-*co*-lactide) with a hydrophilic PEG functionalized with azide groups. The amphiphilic polymer obtained presented azide moieties on the surface for further modification with the alkyne-KGRGDS peptide by CuAAC (Figure 8d) [39]. Due to the presence of RGD-containing peptides, the abovementioned stable and efficient click biofunctionalized polymers showed a peculiar cell response, promoting specific cell adhesion and growth.

## 4. Conclusions

The classical advantages of click chemistry reactions (high yields, simple and mild reaction conditions, easy purification and use of water as the choice solvent) have allowed the conjugation of a number of different biomolecules, thus obtaining large and complex systems with tailored properties. Among these bioconjugate compounds, structures containing the highly recognized RGD tripeptide motif have obtained remarkable interest. The ability to bind selectively to transmembrane proteins on the cell surface and to involve cellular adhesion has allowed RGD to be a useful tool for the functionalization of (bio)materials and for the transfer of particular properties. Selective molecular probes carrying radiolabel atoms or fluorescent moieties have been prepared and used for the identification of specific target cells. In the biopolymer area, new materials able to act as cell attachment and proliferation points have opened the route to new and attractive possibilities of bioconjugation. Undoubtedly that the beneficial effects of click chemistry for the synthesis of biomolecules containing the RGD system will ensure the growth of this area in the future.

## Abbreviations

alanine (Ala, A), arginine (Arg, R), asparagine (Asn, N), aspartic acid (Asp, D), cysteine (Cys, C), glycine (Gly, G), glutamic acid (Glu, E), histidine (His, H), isoleucine (Ile, I), leucine (Leu, L), lysine (Lys, K), methionine, (Met, M), phenylalanine (Phe, F), proline (Pro, P), serine (Ser, S), threonine (Thr, T), tyrosine (Tyr, Y). The symbols in capital letters indicate natural L-aminoacids and the symbols in minuscule letters indicate D-aminoacids.
